# Characteristics associated with self-rated health in the CARDIA study: Contextualising health determinants by income group

**DOI:** 10.1016/j.pmedr.2016.06.001

**Published:** 2016-06-08

**Authors:** Shilpa Nayak, Alan Hubbard, Stephen Sidney, S. Leonard Syme

**Affiliations:** a Department of Public Health and Policy, The Whelan Building, Quadrangle, The University of Liverpool, Liverpool L69 3GB, UK; b School of Public Health, The University of California, Berkeley, Berkeley, USA; c Kaiser Permanente Northern California Division of Research, Oakland, USA

**Keywords:** Self-rated health, Health inequalities, Socioeconomic factors, Health determinants, Classification trees

## Abstract

An understanding of factors influencing health in socioeconomic groups is required to reduce health inequalities. This study investigated combinations of health determinants associated with self-rated health (SRH), and their relative importance, in income-based groups.

Cross-sectional data from year 15 (2000 − 2001) of the CARDIA study (Coronary Artery Risk Development in Young Adults, USA) - 3648 men and women (mean 40 years) - were split into 5 income-based groups. SRH responses were categorized as ‘higher’/‘lower’. Health determinants (medical, lifestyle, and social factors, living conditions) associated with SRH in each group were analyzed using classification tree analysis (CTA).

Income and SRH were positively associated (*p* < 0.05). Data suggested an income-based gradient for lifestyle/medical/social factors/living conditions. Profiles, and relative importance ranking, of multi-domain health determinants, in relation to SRH, differed by income group. The highest ranking variable for each income group was chronic burden-personal health problem (<$25,000); physical activity ($25–50,000; $50–75,000; $100,000 +); and cigarettes/day ($75–100,000). In lower income groups, more risk factors and chronic burden indicators were associated with SRH. Social support, control over life, optimism, and resources for paying for basics/medical care/health insurance were greater (%) with higher income.

SRH is a multidimensional measure; CTA is useful for contextualizing risk factors in relation to health status. Findings suggest that for lower income groups, addressing contributors to chronic burden is important alongside lifestyle/medical factors. In a proportionate universalism context, in addition to differences in intensity of public health action across the socioeconomic gradient, differences in the type of interventions to improve SRH may also be important.

## Highlights

• Health is influenced by interaction of multiple risk and protective factors.
• Classification trees highlight multiple health determinants associated with SRH.
• Combinations and relative importance of risk factors associated with SRH vary by income.
• Differences in intensity and type of action may improve health status across the social gradient.
• In lower income groups addressing both lifestyle and contextual factors appears important.

## 1 Introduction

The socioeconomic gradient in health is well recognized. Knowledge of differences in characteristics associated with good or poor health in socioeconomic groups is important to inform appropriate interventions, and improve health status across the gradient. Health status is a complex construct. The health implications of a single risk factor or exposure may not be universally identical; that is, health status would depend on interaction with coexisting variables, so that different combinations of risk and protective factors produce different outcomes. A solitary focus on single risk factors overlooks the combined impact of these multi-domain influences on health status (Marmot et al., 1998, Ostlin et al., 2005). The WHO Task Force on Research Priorities for Equity in Health called for research studying the “interrelationships between individual factors and social context that increase or decrease the likelihood of achieving and maintaining good health” (Ostlin et al., 2005).

SRH is a common measure of global health status, and an independent predictor of subsequent morbidity and mortality (CDC, 2016a, CDC, 2016b, CDC, 2016c, Idler and Benyamini, 1997, Moller et al., 1996; ONS). For high proportions of populations to report good SRH is in itself an important end point. Studies have identified independent determinants of SRH from diverse domains, including demographic, lifestyle, socio-environmental factors, and physical and mental health status; higher education and income are associated with better SRH status (Franks et al., 2003, Kunst et al., 2005; Mackenbach, 2005; Manderbacka et al., 1999, McFadden et al., 2008, Molarius et al., 2007, Shields, 2008, Shields and Shooshtari, 2001, Singh-Manoux et al., 2006). Adult SRH is also influenced by early-life factors (e.g. social circumstances at birth and school qualifications) (Power et al., 1998). The potential modifying effect of socioeconomic status (SES) on the relationship between objective health and SRH has been explored in earlier studies, with inconsistent findings (Delpierre et al., 2009, Delpierre et al., 2012, Dowd and Zajacova, 2010, Onadja et al., 2013, Singh-Manoux et al., 2007). There is also evidence suggesting SES does not modify the association between SRH and mortality, and that influence of health-related predictors is similar across socioeconomic groups (Burstrom and Fredlund, 2001, McFadden et al., 2009, Smith et al., 2010). Such inconsistencies may in fact result in an underestimation of health inequalities (Delpierre et al., 2009, Dowd and Zajacova, 2010, McFadden et al., 2009, Singh-Manoux et al., 2007). SES may affect expectations of health and risk, the factors considered in assessing subjective health, or their relative weighting. Socioeconomic circumstances can determine the range of factors pertinent to health; we explore this further, in the context of income, in the present study.

In addition to adverse childhood circumstances, a greater prevalence of adverse material circumstances, unhealthy behaviors and psychosocial factors are important in explaining health inequalities (van Lenthe et al., 2004). Lifestyle choices are rooted in socioeconomic context. In targeting factors such as physical exercise, smoking or alcohol consumption, there is value in understanding the concurrent upstream factors that might influence or restrict these choices (Marmot et al., 1998). Meyer et al., for example, found low SES linked to greater neighborhood safety concerns; these were negatively associated with physical activity, which in turn was negatively linked with mental health and SRH (Meyer et al., 2014). Thus, in low SES groups acting primarily on physical activity levels without addressing contextual factors which influence it, may not impact on health status. Mitigation of cumulative adverse effects requires a multi-level and multi-dimensional approach to intervention (Morello-Frosch et al., 2011, Wen et al., 2006).

Given the complexity of the socioeconomic gradient in health, Adler et al. discussed conceptual and methodological issues constraining earlier research on SES and health; one such issue is the limited ability of parametric multivariate regression to capture a large number of multi-domain interrelated variables, and fully unravel the mechanisms that might contribute to the gradient (Adler et al., 1994). Classification tree analysis (CTA), a form of recursive partitioning, provides an alternative approach with several advantages: this non-parametric technique is valuable for studying a complex set of predictor variables, and large sample size; it is data-adaptive, handles high dimensionality, a mixture of data types, and non-standard data structure, while providing insight into the predictive structure of the data (Breiman et al., 1984). Tree-based methods have been used to partition individuals and establish high risk groups by clinical signs and symptoms (Kershaw et al., 2007); they may also uncover interactions potentially overlooked in logistic regression, unless modeled a priori (Forthofer and Bryant, 2000, Lemon et al., 2003, Nelson et al., 1998).

The aim of this study is to apply CTA to investigate combinations of multi-domain health determinants associated with self-rated health (SRH), and conduct an exploratory analysis of their combinations and relative importance in income-based groups. The factors considered represent multiple influences from the social-ecological model of health; a fundamental aim of the study was to contextualize these multi-domain factors, and study their potential joint impact and interactions. It is unclear whether the relative importance of risk factors associated with health status remains the same across income-based groups. We propose these would vary based on interaction of lifestyle choices, psychosocial factors, and living and working conditions, influenced by socioeconomic context. An understanding of these differences is important in planning interventions to improve health status, and reduce income-based health inequalities.

## 2 Methods

This study utilized cross-sectional data collected by the CARDIA longitudinal study (Coronary Artery Risk Development in Young Adults), started in 1985 with a cohort of 5115 men and women aged 18–30 years (1.1% of participants were 17–35 years), recruited in Birmingham, Alabama; Chicago, Illinois; Minneapolis, Minnesota; and Oakland, California. For this study, data were taken from the year 15 follow-up, conducted 2000–2001 (except race/ethnicity - 1985–1986, family history - 1995), through interviewer and self-administered questionnaires, to examine associations between SRH and many health determinants assessed for adults (mean age 40 years) in that year. From 5115 participants at baseline, 3672 were followed-up in year 15; participants with a response for SRH, coded as male or female, were included in the study sample of 3648 participants.

### 2.1 Study variables

Outcome, SRH, was assessed on a 5-point scale: “In general would you say your health is excellent, very good, good, fair or poor?” Responses of poor/fair/good were grouped as ‘lower’ SRH. Responses of very good/excellent were grouped as ‘higher’ SRH, as they were more definite positive statements of better health; respondents may have regarded a response of good, being the centre of a 5-point scale, as a neutral or ‘average’ value. This grouping also resulted in more equal group sizes. In a previous study, fair and good self-ratings of health were associated with higher mortality, so that risk was not associated solely with the poor group, but a gradient was observed. (Idler et al., 1990).

Predictor variables used in the analysis (appendix, Table 1A) represented multiple domains and a range of health determinants based on the social-ecological model of health: age, sex, and hereditary factors; individual lifestyle factors and medical history; social and community influences; living and working conditions (Gebbie et al., 2003, Dahlgren and Whitehead, 1991).

### 2.2 Statistical analyses

The study sample was split into 5 groups based on respondents' total family income. Mantel-Haenszel chi-square test for trend was used to assess the relationship between predictor variables and ordinal income categories. Continuous variables were analysed using the Kruskall-Wallis test.

For each income-based group, CTA was run using all predictor variables, excluding total family income, to segment the group into smaller mutually exclusive subgroups of individuals, and identify predictor variables associated with the outcome measure, SRH. At every node of the tree model formed in the analysis, the sample of individuals was split based on the predictor variable that maximised the goodness of split function, i.e. resulted in the largest decrease in impurity of the prior ‘parent’ node (a node that is split further into subgroups), in terms of distribution of SRH status. A ranking of predictor variables was based upon normalized importance, ranging from 0 to 100, with the variable with the greatest relative measure of importance scored at 100, and other variables scored in the range 0 to 100 (Breiman et al., 1984). Tree growing criteria were set to a minimum ‘parent node’ size of 20 (individuals) and ‘child node’ size of 10 (a child node is a subgroup formed from splitting of a parent node). Data were analysed using IBM SPSS Statistics (SPSS v21).

## 3 Results

Distribution of the study sample by income-based group: under $25,000: 16% (*n* = 578); $25,000–$50,000: 25% (*n* = 911); $50,000–$75,000: 22% (*n* = 791); $75,000–$100,000: 15% (*n* = 527); and $100,000 and over: 22% (*n* = 797) (*N* = 3604; income data missing for 44 participants).

Distribution of study variables by income-based group (Table 1):

SRH: Proportion of ‘higher’ SRH increased with income (*p* < 0.05).

Sex, race/ethnicity and hereditary factors: There was an increasing proportion of males and whites, with higher income, and an inverse income gradient for proportion of respondents with family history of diabetes, stroke, maternal high blood pressure, maternal angina, and maternal heart attack. Higher income groups had higher age respondents (Table 2).

Lifestyle factors and medical history: Medical conditions showed a significant inverse income gradient, except for heart disease; high cholesterol increased with income. The chi-square test was significant (*p* < 0.05) for the relationship between physical activity and income. The lowest income group (<$25,000) had a larger proportion of respondents who rated themselves physically inactive compared to the highest income group ($100,000 plus). Wine consumption increased with income; fast food consumption, beer/hard liquor consumption decreased with income (Table 2).

Social and community influences: In higher income groups, larger fractions of respondents reported good social support and sense of neighborhood cohesion (Table 1).

Living and working conditions: Proportion of home ownership increased with income, and unemployment decreased. There was an inverse income gradient for proportion of respondents reporting difficulty in paying for basics and medical care, and not seeking medical care due to cost. The proportion of respondents with health insurance over the previous two years increased with higher income. Respondents in higher income groups were also more likely to be optimistic for the future, report a sense of control over life events, and be less likely to report feeling helpless in dealing with life problems (Table 1). There were significant associations between 3 indicators of chronic burden and income category by chi-square test for independence (*p* < 0.05) (chronic burden due to serious personal health problem; financial strain; or difficulties in a close relationship).

### 3.1 Classification tree analysis by income-based group

**Fig. 1:**
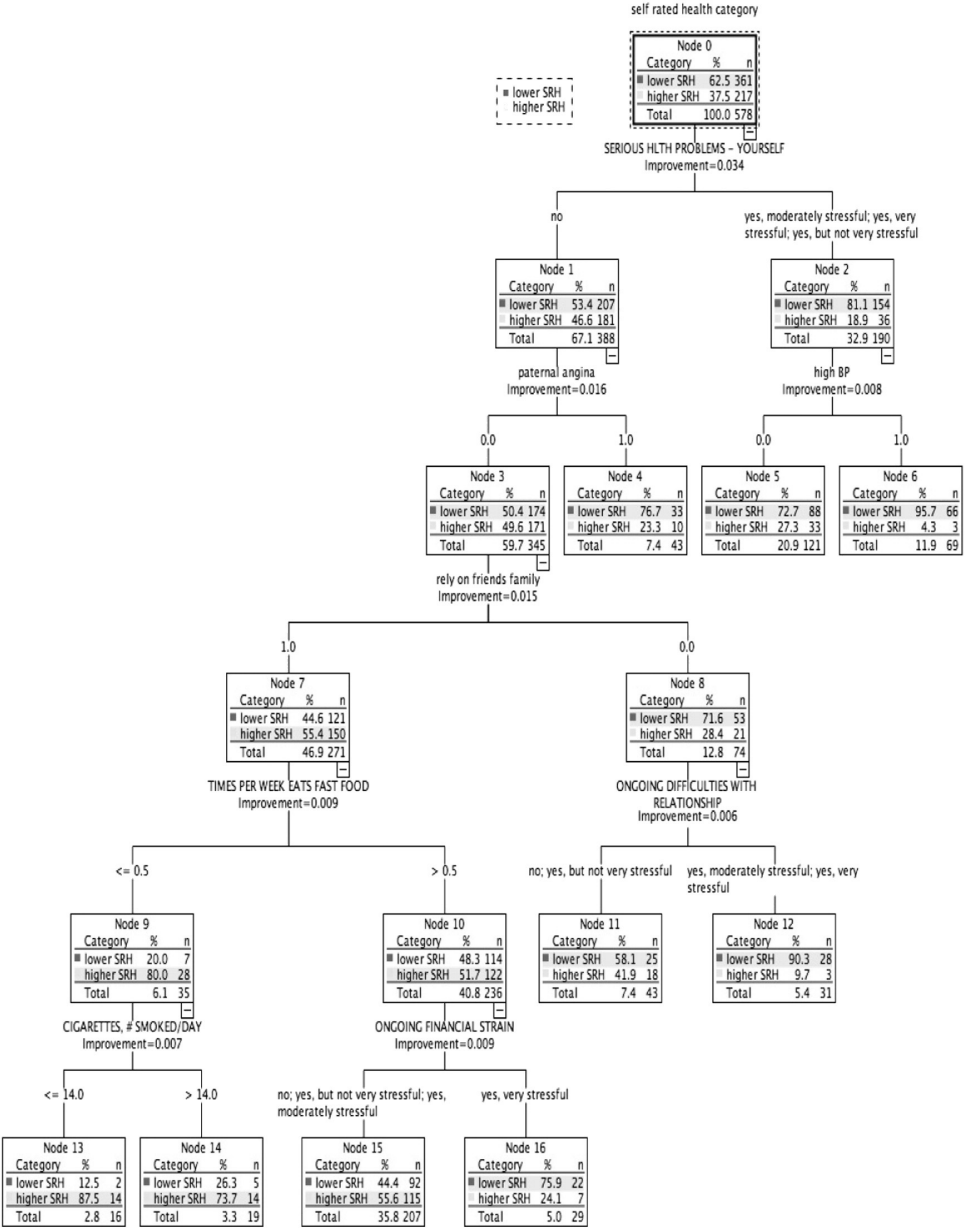
Classification tree for income group under $25,000, CARDIA study, year 15, USA.

Under $25,000 (Fig. 1): Lower SRH: 62.5% (*n* = 361). Three subgroups classed higher (55.6% to 87.5%), and 6 classed lower SRH (58.1% to 95.7%). Associated with SRH status, chronic burden due to serious personal health problem ranked highest. Family history of heart disease; medical conditions (high BP, diabetes, heart disease, mental/nervous/emotional disorder, and depression); all lifestyle factors; ability to rely on friends/family; and all chronic burden indicators were also related to SRH status.

**Fig. 2:**
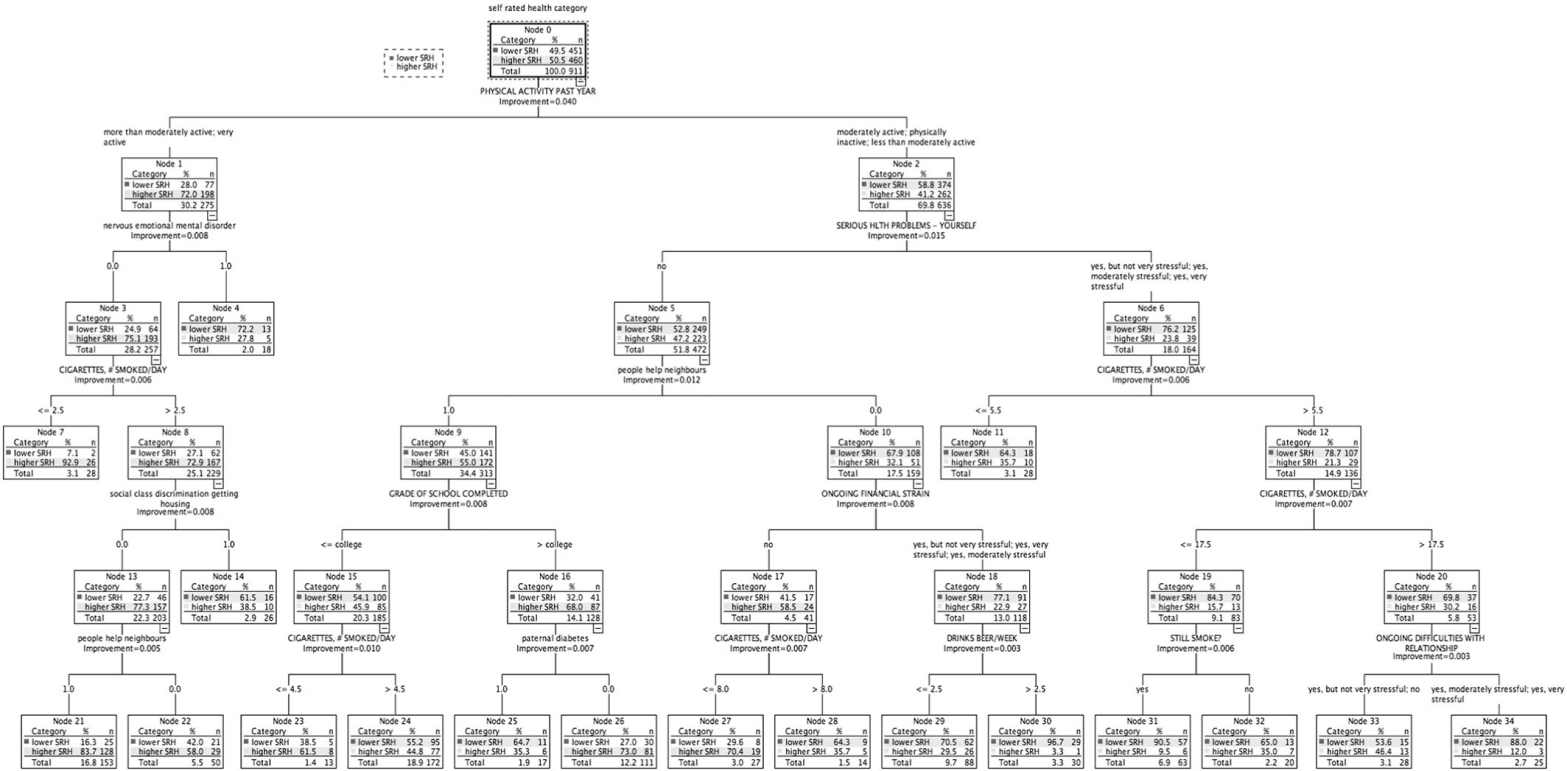
Classification tree for income group $25,000–$50,000, CARDIA study, year 15, USA.

$25,000 to $50,000 (Fig. 2): Higher SRH: 50.5% (*n* = 460). Twelve subgroups classed lower SRH (highest 96.7%), and 6 classed higher SRH (largest 92.9%). Physical activity ranked highest in association with SRH. Nervous/emotional/mental disorder; chronic burden due to (a) serious personal health problem, (b) ongoing financial strain, and (c) close relationship difficulties; cigarettes/day; neighborhood support; social class discrimination getting housing, education; paternal diabetes, and alcohol were all related to SRH status.

**Fig. 3:**
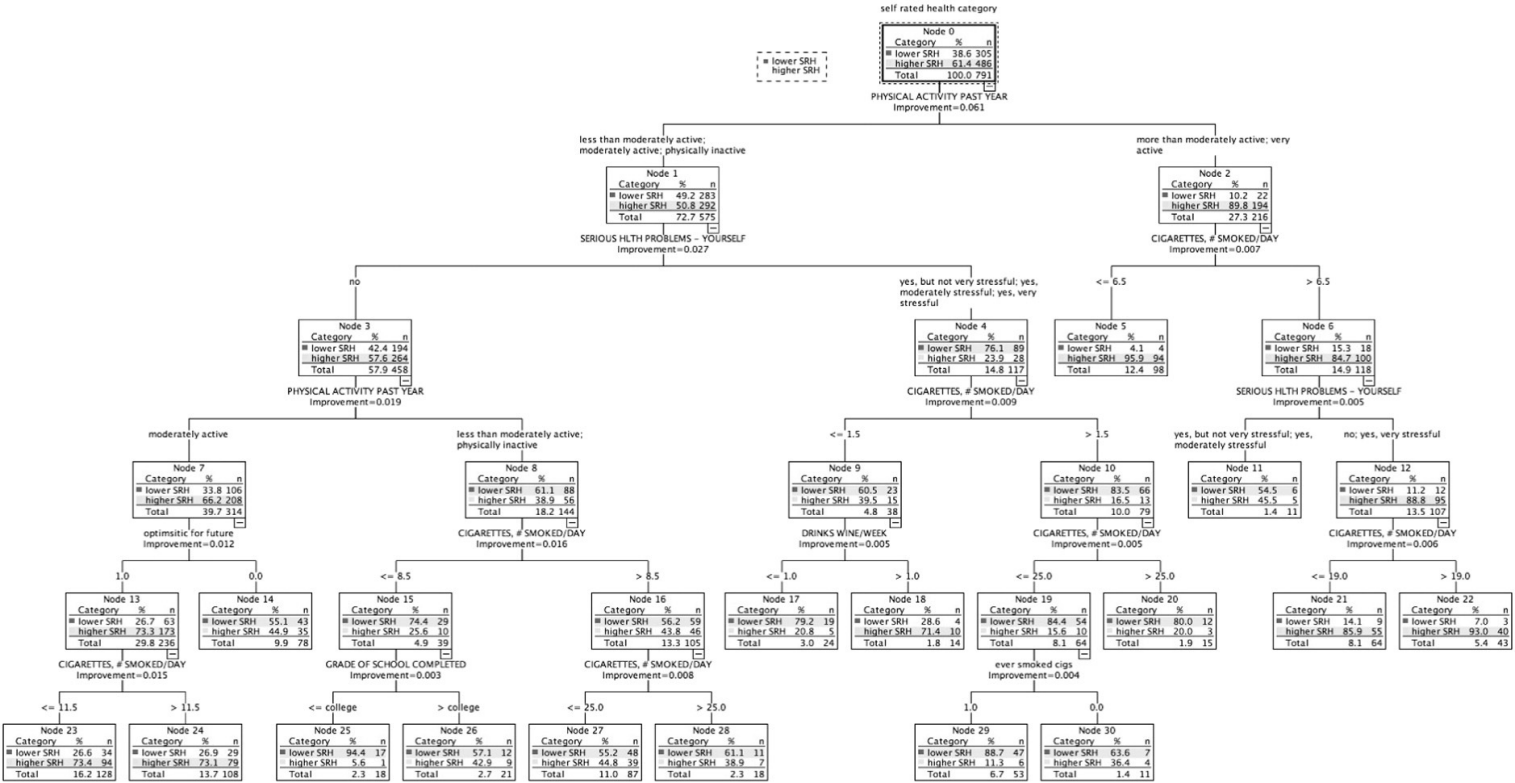
Classification tree for income group $50,000–$75,000, CARDIA study, year 15, USA.

$50,000 to $75,000 (Fig. 3): Higher SRH: 61.4% (*n* = 486). Six subgroups classed higher SRH (largest 95.9%), and 10 classed lower SRH (largest 94.4%). Physical activity was ranked highest in association with SRH (Table 3). Smoking, chronic burden due to serious ongoing personal health problem; optimism for the future, alcohol consumption, and education were also related to SRH status.

**Fig. 4:**
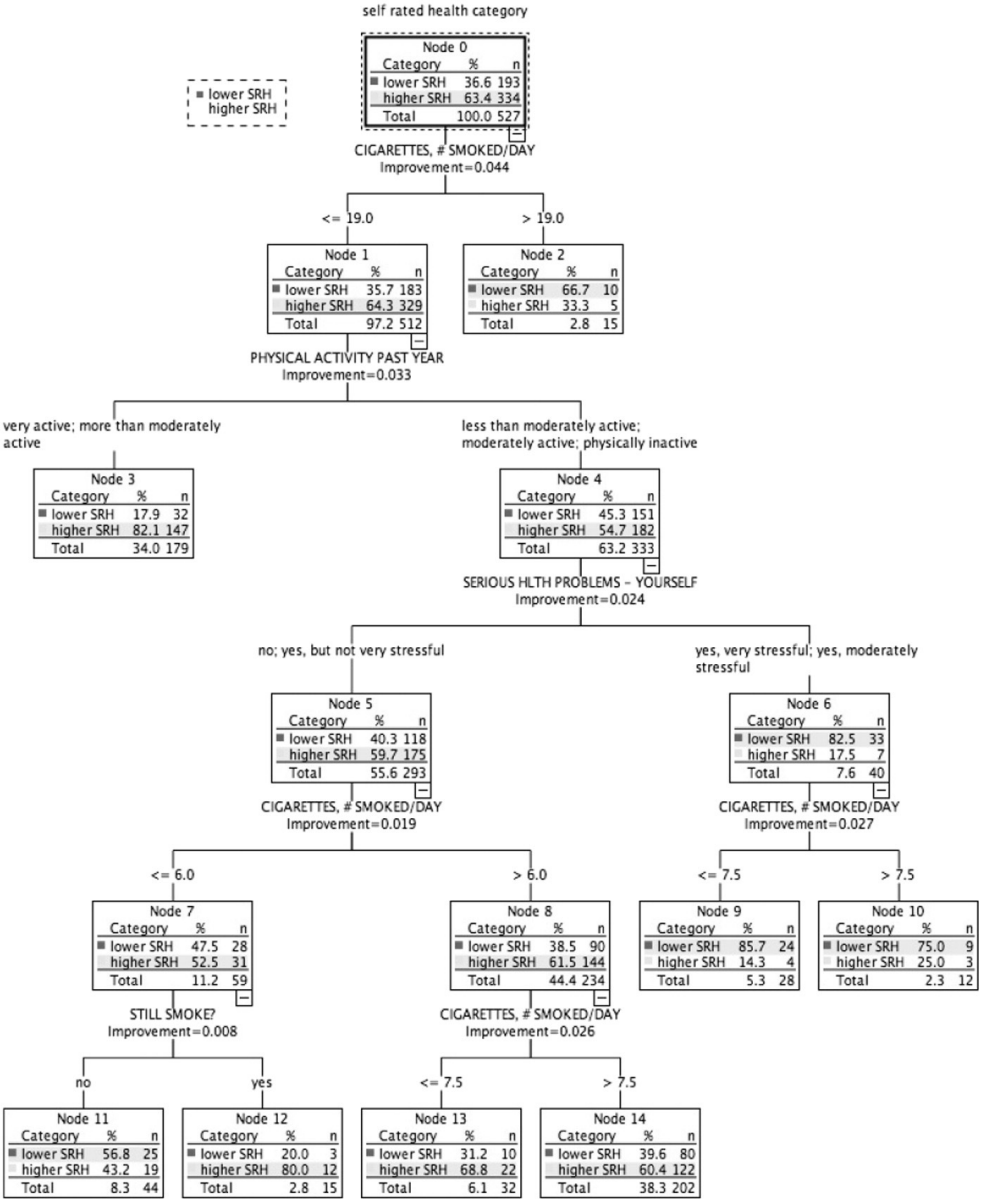
Classification tree for income group $75,000–$100,000, CARDIA study, year 15 USA.

$75,000 to $100,000 (Fig. 4): Higher SRH: 63.4% (*n* = 334). Four subgroups classed lower SRH (highest 85.7%), and 4 classed higher SRH (largest 82.1%). Cigarettes per day ranked highest in association with SRH (Table 3). Physical activity, and chronic burden due to serious personal health problem were also related to SRH status.

**Fig. 5:**
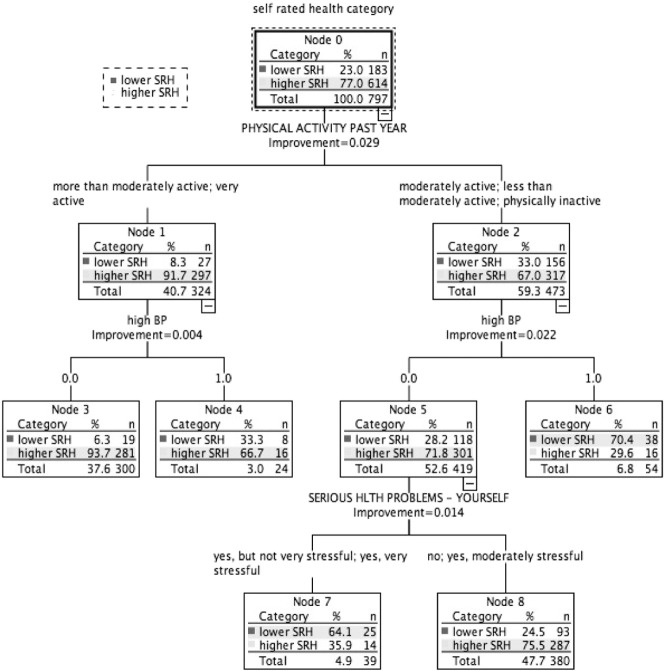
Classification tree for income group $100,000 plus, CARDIA study, year 15, USA.

$100,000 plus (Fig. 5): Higher SRH: 77.0% (*n* = 614). Two subgroups had predominantly lower SRH (highest 70.4%) and 3 had predominantly higher SRH (largest 93.7%). Physical activity was ranked highest in association with SRH (Table 3). High blood pressure and chronic burden due to serious personal health problem were also related to SRH status.

All other multi-domain factors associated with SRH for each income-based group are shown in Table 3, ranked by normalized importance.

Overall estimated misclassification rates based on cross-validation for the tree models were 37% (under $25,000); 38% ($25,000–$50,000); 27% ($50,000–$75,000); 34% ($75,000–$100,000); and 21% ($100,000 and over).

## 4 Discussion

In this study, classification tree analysis reflects SRH as being a multifactorial measure. Consistent with previous studies, prevalence of higher SRH increased with higher income; there was an income gradient for several health determinants relating to lifestyle and medical factors, social and community influences, and living conditions. However, in addition, the results suggest a meaningful variation in the combinations and relative importance of these risk and protective factors associated with health status across the income gradient. Within each income-based group, we also identified smaller subgroups with similar SRH status but associated with differing combinations of risk factors (Fig. 1, Fig. 2, Fig. 3, Fig. 4, Fig. 5). Table 3 highlights the greater range of multi-domain factors linked to SRH status, with lower income. In the lowest income group, all 5 chronic burden indicators were associated with SRH; family history of heart disease, medical and lifestyle factors, and ability to rely on friends/family were also associated with SRH. In the $25–50k income group, 4 of the chronic burden indicators were associated with SRH; neighborhood factors and social support indicators were relevant too, in combination with medical and lifestyle factors, with physical activity highest ranked. For the 2 highest income categories, lifestyle factors (physical activity and smoking) were highest ranked, and there were fewer chronic burden indicators in the model. Based on these results, we suggest that taking account of these differences in risk factor profiles between groups would be important for health promotion; interventions need to be tailored to address priorities based on income or socioeconomic context, and relative importance of interacting multi-domain factors.

Chronic burden from a serious personal health problem was associated with SRH across all income groups with varying relative importance (highest ranked in the lowest income group). In the CARDIA study, this variable relates to the experience of strains over 6 months, and so reflects not simply presence of a medical condition, but associated burden or stress; this may be due to medical costs, access to services, lack of social support in dealing with illness, and the impact on daily life. In populations with illness or disability, resources such as a sense of high mastery, greater self-esteem or social support have been shown to associate with better SRH (Bosworth et al., 1999, Cott et al., 1999). Social support, positive social relationships, optimistic outlook on life, perceived control over life outcomes, and sense of purpose and direction in life, have been identified as health protective factors; psychosocial factors also influence positive health behaviors (WHO, 2002). In the study sample, lower income groups had additional sources of chronic burden (e.g. from financial strain; difficulties in close relationships; and job or ability to work) associated with SRH. Higher income groups had higher prevalence of individuals reporting: good social support and sense of neighborhood cohesion; availability of health insurance, resources for basics and medical care; optimism; sense of control; and a lower prevalence of feeling helpless in dealing with life problems. This suggests a possible protective or buffering role of these factors from the associated burden or stress of health problems.

Limitations to the present study include the cross-sectional study sample, the self-reported nature of predictor variables, household income unadjusted for occupancy, and potential of health selection in use of income as the marker of SES. However, as choice of socioeconomic indicator depends on the postulated mechanisms by which it affects health (Lynch and Kaplan, 2000), the use of income is justified, as the focus is on SES in terms of disparities in material resources, and access to resources. Higher versus lower SRH status is compared in this initial study, though further detail, and more complex tree models, would be produced by maintaining 5 response categories for SRH status. Classifying responses of good, fair and poor into one category may be viewed as a limitation if health determinants associated with good SRH are more similar to very good/excellent health in this sample. It is not possible to make further assumptions regarding importance of individual ranked factors based on a single tree classification, and no inference exists for the difference of the variable importance ranking between income groups. This is an exploratory data analysis, and further research is needed to confirm the importance of these factors. However, for each income-based group, the normalization is used to obtain comparable measures of importance that are scale-free, and thus allow a sensible ranking of the predictor variables. This is in order to get an idea of relative importance of the factor in relation to the outcome; this has relevance for prioritizing and targeting action to improve health status.

A broad range of variables is included in the analysis as SRH is influenced by several different factors, and the analytic method used can handle this set of predictors. Some variables that are correlated could be eliminated further by additional investigation, though correlation could also vary by income-based subgroup. Even so, the analytic approach used here is a data-adaptive method that is particularly useful in such cases, when there are a large number of potential explanatory factors, also allowing the most important set of predictors among a large set of candidates to be identified. Recognizing that these are exploratory analyses, it may be inferred that in considering the design of surveys or interventions related to self-reported health, priority could be given to consideration of factors impacting chronic burden related to serious personal health problem (e.g., access to medical care, medical insurance, and lack of support) in low-income individuals, since this is the top node in the tree model. For upper-income individuals, priority could be given to consideration of factors affecting physical activity since this is the top node in the tree model for upper-income individuals.

Contextualising risk factors, by considering the clustering and joint impact of different health determinants, is important for action on the “fundamental factors that put people at risk of risks” (Link and Phelan, 1995). This concept is analogous to the framework used by infectious disease epidemiologists, which considers the agent, host, and susceptibility, or environment. Differential impacts on health occur also because low-income groups are more likely to be exposed to multiple hazardous risk factors simultaneously, and a convergence of environmental hazards and social stressors, which contribute to poor health outcomes, and inequalities (Dahlgren and Whitehead, 2007, Marmot et al., 1998, Morello-Frosch et al., 2011). High-income groups have more resources providing protection from disease and its consequences (Link and Phelan, 1995). Therefore, a health promotion intervention appropriate for one group is not necessarily optimal for another.

Factors such as physical activity and smoking were important for all income groups in our study. To improve health status for lower income groups, also addressing factors contributing to chronic burden, mental illness, neighborhood problems, and lack of social support may be beneficial, and in turn have an effect on lifestyle factors. The analysis unifies multidomain and multilevel factors; the results imply the need for collaborative approaches to improve SRH. Both policy and population-level public health interventions are required to address upstream health determinants, alongside medical and health promotion efforts tackling individual medical or lifestyle factors.

Friel et al. (2005) applied CTA, to study profiles (socio-demographic, socioeconomic factors and health-related lifestyle behaviors) of adults complying with fruit and vegetable dietary recommendations, based on the rationale that food choice is complex and influenced by economic, social and environmental context. Relative importance of social characteristics in predicting fruit and vegetable consumption differed by gender; these results were considered to have implications for setting dietary strategies and policy. BeLue et al. applied classification and regression tree analysis to study obesity-related risk profiles in a sample of US adolescents (BeLue et al., 2009). Obesity-related risk and protective factors differed among sociodemographic groups, and in their relative importance to adolescent overweight status. These results, and those from the present study, demonstrate that in terms of public health intervention, often “one size does not fit all”; improving health requires a multi-level and multi-dimensional approach to intervention, which regards the complexity and diversity of risk factor profiles in different subgroups (BeLue et al., 2009, Morello-Frosch et al., 2011, Wen et al., 2006).

Adler and Stewart's review discussed the eras though which research on SES and health has progressed (Adler and Stewart, 2010). The first model of a threshold effect between poverty and health was refined following evidence of a graded association. Subsequent eras studied mechanisms linking SES and health, and considered multilevel influences, and most recently, interactions among factors. This study utilizes a CTA approach to investigating the factors associated with SRH in income-based groups, and adds to the work on the joint impact of health determinants on SRH. Multivariate logistic regression models may demonstrate only the average relationship between predictor and outcome over the population. CTA is a useful segmentation technique to suggest population subgroups that might have homogenous risks of an outcome, and identify the relative importance of associated risk and protective factors for further inquiry (Forthofer and Bryant, 2000).

The Marmot report, Fair Society Healthy Lives (on evidence-based strategies for reducing health inequalities in England) recommended proportionate universalism to reduce the steepness of the socioeconomic gradient in health (Marmot, 2010). This requires a universal approach to public health action but with a scale and intensity proportionate to level of disadvantage. Our study results suggest potentially important differences in factors associated with SRH among income-based groups. In the context of a proportionate universalism approach to reducing health inequalities, the findings imply that as well as differences in the intensity of public health action required the gradient, differences in the type of actions to improve SRH may also be important.

The following is the supplementary data related to this article.

Table 1A Predictor variables used in analysis - method of assessment in CARDIA questionnaires and variable format. Table 1A

## Human subjects statement

The Coronary Artery Risk Development in Young Adults (CARDIA) study was approved by the institutional review boards at all study sites including Northwestern University, University of Alabama at Birmingham, University of Minnesota, and Kaiser Permanente. Study approval was granted by the Committee for Protection of Human Subjects at the University of California, Berkeley.

## Contributors

SN devised the original idea for the study, conducted data analysis and wrote the preliminary draft of the paper. SLS contributed to study conception. AH contributed to data analysis, and AH and SS to interpretation of data. All authors developed and revised the manuscript and approved the final version.

## Conflict of interest statement

The authors declare that there are no conflicts of interest.

## Figures and Tables

**Table 1:** Distribution of selected predictor variables by income-based group and Mantel Haenszel chi-square test for trend, the CARDIA study, year 15, USA.

Variable	Income categories N (% within income group)					Total	p value^a^
<$25,000	$25,000–$50,000	$50,000–$75,000	$75,000–$100,000	$100,000 plus
*Self-rated health [count (%within income category)]*							
Lower	361 (62.5)	451 (49.5)	305 (38.6)	193 (36.6)	183 (23.0)	1493	< 0.05
Higher	217 (37.5)	460 (50.5)	486 (61.4)	334 (63.4)	614 (77.0)	2111

*Sex, race/ethnicity and family history [count (%within income category)]*							
Male	215 (37.2)	392 (43.0)	327 (41.3)	253 (48.0)	403 (50.6)	1590	< 0.05
Female	363(62.8)	519 (57.0)	464 (58.7)	274 (52.0)	394 (49.4)	2014
Hispanic^b^	4 (0.7)	3 (0.3)	2 (0.3)	0 (0)	2 (0.3)	11	< 0.05^c^
Black	431 (74.6)	529 (58.1)	344 (43.5)	210 (39.8)	167 (20.9)	1681
White	143 (24.7)	379 (41.6)	445 (56.3)	317 (60.2)	628 (78.8)	1912
Maternal diabetes	97 (20.6)	120 (15.2)	109 (15.4)	53 (11.4)	54 (7.6)	433	< 0.05
Maternal high blood pressure (BP)	246 (55.0)	348 (47.5)	294 (45.2)	190 (45)	226 (34.1)	1304	< 0.05
Maternal stroke	52 (10.8)	49 (6.1)	54 (7.6)	21 (4.4)	27 (3.8)	203	< 0.05
Maternal angina	45 (10.0)	72 (9.5)	52 (7.7)	47 (10.5)	36 (5.2)	252	< 0.05
Maternal heart attack	59 (12.4)	64 (8.0)	40 (5.6)	29 (6.2)	23 (3.3)	215	< 0.05
Paternal diabetes	58 (15.6)	108 (16.1)	78 (12.5)	57 (13.1)	83 (12.3)	384	< 0.05
Paternal high BP	163 (48.4)	265 (45.4)	250 (43.8)	186 (48.3)	272 (44.8)	1136	> 0.05
Paternal stroke	46 (12.2)	80 (11.6)	64 (9.9)	44 (10.0)	43 (6.3)	277	< 0.05
Paternal angina	58 (16.7)	93 (15.3)	77 (13.3)	65 (16.3)	119 (18.8)	412	> 0.05
Paternal heart attack	78 (20.9)	149 (21.5)	108 (16.6)	102 (23.1)	143 (21.2)	580	> 0.05

*Lifestyle factors and medical history [count (%within income category)]*							
High BP	153 (26.7)	162 (18)	113 (14.4)	90 (17.2)	78 (9.9)	596	< 0.05
High cholesterol	80 (14.4)	151 (17.1)	142 (18.3)	93 (18.1)	163 (20.7)	629	< 0.05
Heart disease	70 (12.3)	97 (10.8)	90 (11.5)	52 (10.1)	94 (11.9)	403	> 0.05
Diabetes	42 (7.4)	61 (6.8)	39 (4.9)	31 (5.9)	33 (4.1)	206	< 0.05
Transient ischemic attack/stroke	10 (1.7)	4 (0.4)	10 (1.3)	4(0.8)	1 (0.1)	29	< 0.05
Asthma	84 (14.7)	114 (12.6)	94 (11.9)	61 (11.6)	86 (10.8)	439	< 0.05
Chronic bronchitis	36 (6.3)	59 (6.5)	31 (3.9)	16 (3.0)	17 (2.1)	159	< 0.05
Liver disease	27 (4.7)	18 (2.0)	16 (2.0)	10 (1.9)	17 (2.1)	88	< 0.05
Kidney disease	37 (6.4)	48 (5.3)	59 (7.5)	24 (4.6)	61 (7.7)	229	> 0.05
Cancer	22 (3.8)	24 (2.6)	20 (2.5)	22 (4.2)	30 (3.8)	118	> 0.05
Epilepsy	22 (3.8)	9 (1.0)	8 (1.0)	7 (1.3)	6 (0.8)	52	< 0.05
Thyroid disease	25 (4.4)	36 (4.0)	45 (5.7)	30 (5.7)	41 (5.2)	177	> 0.05
Nervous emotional or mental disorder	81 (14.1)	66 (7.3)	52 (6.6)	34 (6.5)	33 (4.2)	266	< 0.05
Depression	121 (21.1)	152 (16.9)	122 (15.5)	74 (14.1)	97 (12.3)	566	< 0.05
Physical activity	61 (10.6)	69 (7.6)	46 (5.8)	28 (5.3)	28 (3.5)	232	< 0.05^d^
1 inactive
2	89 (15.5)	148 (16.3)	163 (20.7)	99 (18.8)	113 (14.2)	612
3	262 (45.5)	418 (45.9)	364 (46.1)	220 (41.7)	331 (41.6)	1595
4	78 (13.5)	140 (15.4)	115 (14.6)	107 (20.3)	187 (23.5)	627
5 very active	86 (14.9%)	135 (14.8)	101 (12.8)	73 (13.9)	137 (17.2)	532
Current smoker	248 (76.1)	244 (63.0)	136 (43.7)	70 (39.3)	87(34.0)	785	< 0.05

*Social and community influences [count (%within income category)]*							
Friends and family care	533 (92.2)	883 (96.9)	786 (99.4)	522 (99.2)	790 (99.1)	3514	< 0.05
Can rely on friends and family	444 (76.8)	768 (84.3)	706 (89.3)	485 (92.2)	739 (92.7)	3142	< 0.05
People help neighbors	352 (60.9)	609 (66.8)	605 (76.5)	406 (77.0)	657 (82.4)	2629	< 0.05
Close knit neighborhood	214 (37.0)	337 (37.0)	324 (41.0)	234 (44.4)	416 (52.2)	1525	< 0.05
Can trust neighbors	213 (36.9)	453 (49.7)	477 (60.3)	346 (65.7)	606 (76.1)	2095	< 0.05
Neighbors get along	307 (53.1)	602 (66.1)	612 (77.4)	414 (78.6)	669 (83.9)	2604	< 0.05
Neighbors share values	191 (33.0)	367 (40.3)	389 (49.2)	298 (56.5)	507 (63.5)	1752	< 0.05

*Living and working conditions [count (%within income category)]*							
Own home	183 (31.7)	510 (56.0)	610 (77.1)	452 (85.8)	729 (91.5)	2484	< 0.05
Unemployed	158 (27.4)	63 (6.9)	36 (4.6)	28 (5.3)	38 (4.8)	323	< 0.05
Hard to pay for basics	295 (51.1)	245 (27.0)	111 (14.1)	38 (7.2)	21 (2.6)	710	< 0.05
Hard to pay for medical care	300 (52.0)	243 (26.8)	93 (11.8)	32 (6.1)	33 (4.1)	701	< 0.05
Health insurance coverage past 2 years	371 (64.3)	771 (84.6)	735 (92.9)	507 (96.2)	774 (97.1)	3158	< 0.05
Did not seek medical care last 2 years due to cost	134 (23.2)	112 (12.3)	50 (6.3)	22 (4.2)	24 (3.0)	342	< 0.05
Control over events	417 (72.1)	746 (81.9)	687 (86.9)	462 (87.8)	735 (92.2)	3047	< 0.05
Helpless in dealing with life problems	105 (18.2)	98 (10.8)	58 (7.3)	26 (4.9)	28 (3.5)	315	< 0.05
Optimistic for future	359 (62.1)	619 (67.9)	563 (71.2)	403 (76.5)	615 (77.2)	2559	< 0.05

a p value for chi-square test for trend: linear-by-linear association.

b CARDIA was designed to be a biracial cohort, however, some information on ethnicity was collected at baseline, and this is reflected by the 11 participants classified in the study as Hispanic.

c 5 cells (33.3%) have expected count less than 5. The minimum expected count is 1.61.

d Chi-square test reported.

**Table 2:** Kruskall-Wallis test for continuous variables, the CARDIA study, year 15, USA.

Income categories	Age *p* < 0.05		Fast food meals/week *p* < 0.05		Wine drinks per week *p* < 0.05		Beer drinks per week *p* < 0.05		Liquor drinks per week *p* < 0.05	
N	Mean rank	N	Mean rank	N	Mean rank	N	Mean rank	N	Mean rank
1 Under $25k	578	1721.01	527	1652.03	392	1272.48	393	1639.52	393	1545.03
2 $25k to $50k	911	1713.74	843	1744.65	675	1313.49	675	1432.85	675	1441.74
3 $50k to $75k	791	1781.06	712	1669.79	626	1350.84	626	1336.62	626	1355.20
4 $75k to $100k	527	1830.26	489	1568.58	427	1425.27	427	1365.51	427	1430.28
5 $100k +	797	1965.98	683	1462.08	730	1675.94	730	1416.75	730	1405.57
Total	3604		3254		2850		2851		2851	

**Table 3:**
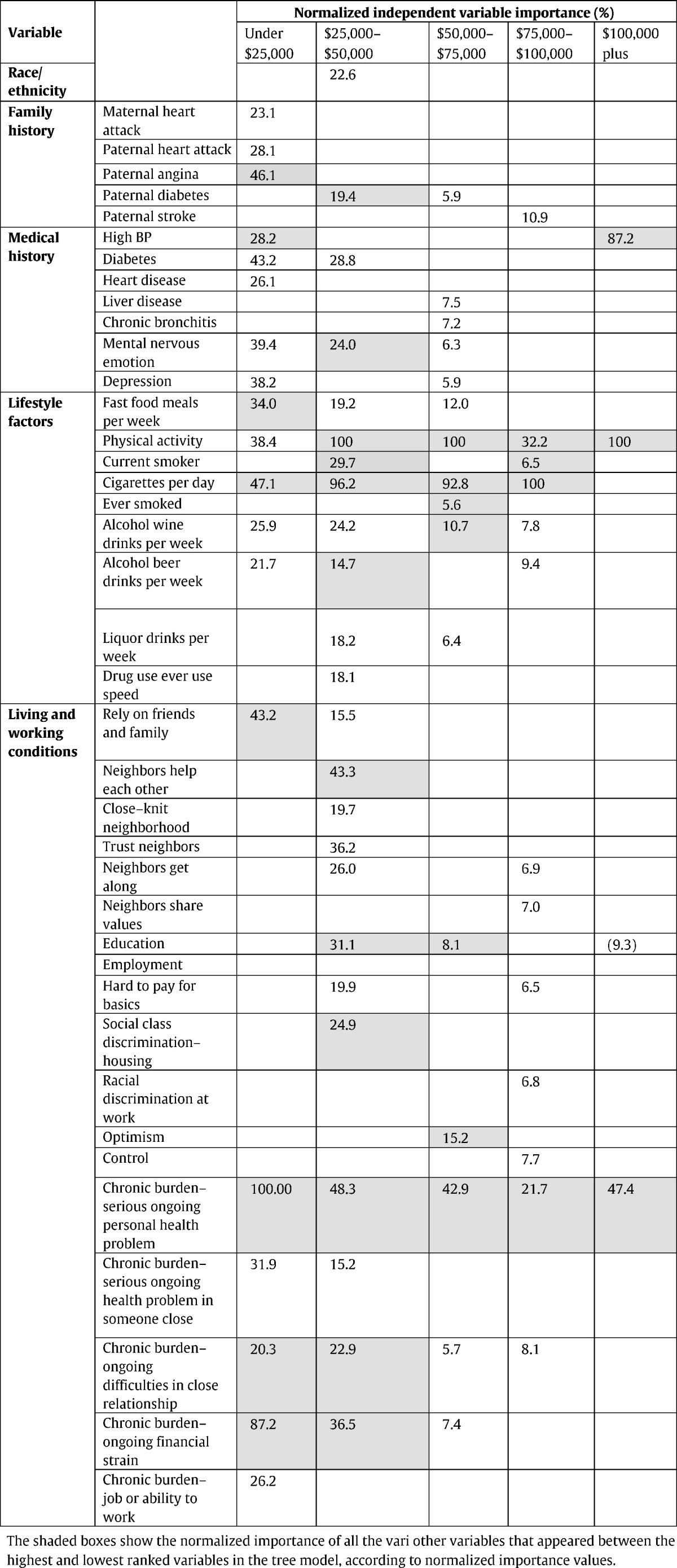
Combinations of health determinants and variable ranking associated with self-rated health by income-based group, the CARDIA study, year 15, USA.
