# Supplementary material for: Characteristics associated with self-rated health in the CARDIA study: Contextualising health determinants by income group
Source: Prev Med Rep. 2016 Jun 8;4:199–208. doi: 10.1016/j.pmedr.2016.06.001 (PMC4929073; doi:10.1016/j.pmedr.2016.06.001)
Supplement: Table 1A — Predictor variables used in analysis - method of assessment in CARDIA questionnaires and variable format. [file mmc1.doc]

**Appendix**

Table 1A: Predictor variables used in analysis - method of assessment in CARDIA questionnaires and variable format

| **Variable** | **Variable format for study analysis** |
| --- | --- |
| **Age, sex and hereditary factors** | |
| **Age** | Age in years |
| **Sex** | Male or Female |
| **Race/ethnicity** | Hispanic, black (not Hispanic), white, not Hispanic |
| **Family History** | History of maternal or paternal diabetes, high blood pressure, stroke, angina, heart attack |
| **Individual lifestyle factors and medical history** | |
| **Medical history – presence of disease** | History of disease for each condition.  high blood pressure; high blood cholesterol; heart disease; asthma; chronic bronchitis; emphysema; diabetes; liver disease; kidney disease (excluding nephritis or glomerulonephritis); cancer or malignant tumour; HIV; stroke or TIA (transient ischemic attack); multiple sclerosis; epilepsy (seizures); nervous / emotional or mental disorder; depression |
| **Diet** | Number of times per week that breakfast, lunch, or dinner eaten out in fast food restaurant such as McDonald's, Burger King, Wendy's, Arby's, Pizza Hut, or Kentucky Fried Chicken |
| **Physical activity** | 5 point rating of physical activity compared to other people of same age and sex during the past year? (1=physically inactive to 5=very active) |
| **Smoking / Tobacco** | History, for at least 3 months, of being regular cigarette smoker (at least 5 per week almost every week)  Still smoke cigarettes regularly (at least 5 per week almost every week)  Number of cigarettes smoked per day on average (1 pack=20 cigarettes) (continuous variable) |
| **Alcohol** | Number of drinks per week of wine (about a 5oz. glass).  Number of drinks per week of beer (1 beer is a 12 oz. glass, can, or bottle).  Number of drinks per week of hard liquor (each shot of 1½oz. counted as 1 drink). |
| **Illicit drug use** | History of drug use for ever using:  marijuana / crack / other forms of cocaine that are not crack (including powder, free base, and coca paste) / amphetamines ("Speed" or "Uppers") / opiates for non-medical reasons (Heroin, Dilaudid, Morphine, Demerol)? |

Table 1A (cont.)

| **Social and community influences** | |
| --- | --- |
| **Social support / network (“feeling that family friends really care”)** | Family members or friends are perceived to care  Can rely on family members or friends if need to talk about worries. |
| **Sense of close knit neighborhood, neighborhood cohesion** | In thinking about the neighborhood in which you live:  People willing to help their neighbors / Live in close-knit neighborhood.  People in the neighborhood can be trusted.  People in the neighborhood generally get along with each other.  People in the neighborhood share the same values |
| **Living and working conditions** | |
| **Education** | Highest grade (or year) of regular school completed?  01-08=elementary school  09-12=High School  13-16=College  17-20+=Graduate School |
| **Income** | Total combined family income for the past 12 months?  1 = Less than $5,000  2 = $5,000 - $11,999  3 = $12,000 - $15,999  4 = $16,000 - $24,999  5 = $25,000 - $34,999  6 = $35,000 - $49,999  7 = $50,000 - $74,999  10=$75,000 - $99,999  11=$100,000 and greater |
| **Housing - rent or own house** | Own home versus rented, occupied or other |

Table 1A (cont.)

| **Employment - working versus unemployed** | Unemployed status |
| --- | --- |
| **Control & adequacy of resources (“how hard is it to pay for basics”)** | Hard to pay for basics  Hard to pay for medical care |
| **Medical insurance** | Always had health insurance or other coverage for medical care in the past two years.  Covered by health insurance like Blue Cross/Blue Shield or participation in an HMO; health insurance obtained through an employer, union, or school.  Self-insured |
| **Access to health services** | Categorical indicator variables:  Did not seek medical care in past 2 years due to cost  Has been hard overall getting health services  1=’hard’ (very /fairly)  0=’not hard’ (not too hard/not hard at all) |
| **Experience of discrimination due to:**  **Gender**  **Race/ethnicity or colour**  **Socioeconomic position or social class** | Experience of discrimination due to gender / race-ethnicity or color / socioeconomic position or social class for each setting:  At school  Getting a job  Getting housing  At work  At home  Getting medical care  On the street or in a public setting |

Table 1A (cont.)

| **Some type of on-going chronic burden** | Experienced strains for longer than 6 months due to  Serious ongoing health problem (yourself).  Serious ongoing health problem (someone close to you).  Ongoing difficulties with your job or ability to work  Ongoing financial strain  Ongoing difficulties in a relationship with someone close to you  1=No  2=Yes, but not very stressful  3=Yes, moderately stressful  4=Yes, very stressful |
| --- | --- |
| **Optimism for the future** | Have no control over the things that happen  Feel helpless in dealing with the problems of life  Always optimistic about future |
